# Carbohydrate-Containing Marine Compounds of Mixed Biogenesis

**DOI:** 10.3390/md19120694

**Published:** 2021-12-06

**Authors:** Valentin A. Stonik, Natalia V. Ivanchina

**Affiliations:** G.B. Elyakov Pacific Institute of Bioorganic Chemistry, Far Eastern Branch, Russian Academy of Sciences, Pr. 100 Let Vladivostoku, 159, 690022 Vladivostok, Russia

Marine natural compounds, containing rare and enzymatically-modified monosaccharide residues [1] and/or having fragments of both carbohydrate and non-carbohydrate nature, attract attention with their unusual structures and biological activities [2,3,4]. There are biopolymers and low molecular weight products among them. Not only enzymes of carbohydrate biosynthesis, but also sets of other enzymes, including oxidases, isomerases, sulfatases, and methylases, are involved in the biosynthesis of sugar chains in marine glycosides and polysaccharides. In addition to steroid and triterpene saponins, widely distributed in some classes of marine invertebrates, other glycosides, with isoprenoids, alkaloids, and fatty alcohols, such as aglycones, which are formed by mixed biogenesis, have also been found in marine organisms.

At present, we are operating as editors of the Special Issue “Carbohydrate-containing marine compounds of mixed biogenesis”, https://www.mdpi.com/journal/marinedrugs/special_issues/Carbohydrate-containing (accessed on 12 November 2021). This Special Issue contains eight original papers related to different interesting topics concerning isolation, structure elucidation, and biomedical properties of the corresponding compounds and two reviews with comprehensive analyses of literature data on different classes of marine natural compounds. Herein, in the following sections, we provide a short overview of the research findings by the authors of this Special Issue.

Several papers were dedicated to marine glycosides, natural products of mixed biogenesis, particularly characteristic of starfishes and holothurians (sea cucumbers) (the phylum Echinodermata). For instance, Silchenko et al. [5] reported the results of studies on six new triterpene tetra-, penta-, and hexaosides, namely chitonoidosides A, A_1_, B, C, D, and E, from the Far Eastern sea cucumber *Psolus chitonoides*. These compounds, containing one or two sulfate groups, were isolated from the animals and collected near Bering Island (Commander Islands) from the depth of 100–150 m. Three of them, chitonosides A, B, and E, have unprecedented aglycones with 18(20)-ether bond and therefore lack the 18(20)-lactone found in the majority of earlier studied holothurian glycosides. The cytotoxic effect of chitonoidoside D against human erythrocytes, adenocarcinoma HeLa, colorectal adenocarcinoma DLD-1, and leukemia promyeloblast HL-60 human cancer cells was shown to be the most significant in this series.

The review paper “Asterosaponins: structures, taxonomic distribution, biogenesis and biological activities” by a group of the Russian scientists [6] summarized the literature data concerning this class of steroid oligoglycosides and gave a list, as complete as possible, of all these secondary metabolites with structures known to date. This review includes principal information about their taxonomic distribution, findings in starfish collected in different geographical areas and depths, some chemical properties, biological activities, and functions. Structures and properties of some natural compounds, closely related to classical asterosaponins, were also discussed.

In the original paper of Makarieva et al. [7], isolation, structure, and antifungal activity of oceanalin B, an unusual natural compound of unexpected mixed biogenesis from the lyophilized marine sponge *Oceanapia* sp., were reported. Oceanalin B is substituted with tetrahydroisoquinoline α,ω-bipolar sphingoid glycoside. It presents particular interest due to its unusual structure and biological activity. Furthermore, this compound demonstrates an inhibitory effect against the pathogenic fungus *Candida glabrata*, with a MIC of 25 μg/mL.

In a review article by Stonik and Kolesnikova [8], information concerning structural diversity, biological activities, and syntheses of rare groups of malabaricane and isomalabaricane triterpenoids, including some glycosides derived from them, was given. Representatives of these groups were found in higher and lower terrestrial plants, fungi, and marine sponges. Evolution of these triterpenoids provided a variety of rearranged, oxidized, and glycoconjugated metabolites. These natural compounds have attracted attention, not only due to their biogenetic origin, but also their biological activity, which is particularly extremely high cytotoxicity against tumor cells.

Marine polysaccharides and their semi-synthetic derivative have become the objects of research, described in another series of papers. Therefore, the structure of the capsular polysaccharide from the marine psychrophilic Gram-negative bacterium *Polysyncraton marincola* KMM 277^T^ and its effect on the viability and colony formation of human acute promyelocytic leukemia HL-60 cells were reported in a paper by Kokoulin et al. [9]. It was found that the polysaccharide consists of branched hexasaccharide repeating units containing two 2-N-acetyl-2-deoxy-d-galacturonic acids and one of each of 2-N-acetyl-2-deoxy-d-glucose, d-glucose, d-ribose, and 7-N-acetylamino-3,5,7,9-tetradeoxy-5-N-[(R)-2-hydroxypropanoylamino]-l-glycero-l-manno-non-2-ulosonic acid. This was the first research to find a pseudaminic acid decorated with lactic acid residue in polysaccharides. The isolated capsular polysaccharide significantly reduced the viability and colony formation of tumor HL-60 cells.

Sigida et al. [10] studied the moderately halophilic bacterial strain *Salinivibrio* sp. EG9S8QL, isolated from saline mud (Emisal Salt Company, Lake Qarun, Fayoum, Egypt). The isolated lipopolysaccharide was studied by sugar analysis, along with ^1^H and ^13^C NMR spectroscopy, including ^1^H,^1^H COSY, TOCSY, ROESY, ^1^H,^13^C HSQC, and HMBC experiments. It was found to be composed of linear tetrasaccharide repeating units of the following structure: →2)-β-Manp4Lac-(1→3)-α-ManpNAc-(1→3)-β-Rhap-(1→4)-α-GlcpNAc-(1→, where Manp4Lac is 4-O-[1-carboxyethyl]mannose.

A novel bacterial exopolysaccharide EPS364 with a molecular weight of 14.8 kDa was obtained from *Vibrio alginolyticus* 364 from a deep-sea cold seep of the South China Sea by Wang et al. [11]. It consisted of mannose, glucosamine, gluconic acid, galactosamine, and arabinose, with a molar ratio of 5:9:3.4:0.5:0.8. EPS364 exhibited a significant antitumor activity, inducing apoptosis, dissipation of the mitochondrial membrane potential (MMP), and generation of reactive oxygen species (ROS) in Huh7.5 liver cancer cells. Proteomic and quantitative real-time PCR analyses indicated that EPS364 inhibits cancer cell growth and adhesion via targeting the FGF19-FGFR4 signaling pathway.

Dong et al. [12] showed that the sulfated polysaccharides extracted from the seaweed *Porphyra haitanensis* possesses by antioxidant activity in the concentration range of 1–5 mg/mL. In the experiments, when simulating gastric juice and alpha amylase utilization in vitro, it was indicated that PHPs can better resist digestion of alpha amylase and have better resistance than fructooligosaccharide. This natural product has potential prebiotic activity and demonstrates the potential for use in the food and cosmetic industries.

Malyarenko et al. [13] examined the anticancer effect of sulfated derivative of laminaran from the brown alga *Alaria angusta* and polyhydroxysteroid glycosides protolinckiosides A, B, and linckoside L1 from the starfish *Protoreaster lincki* against colorectal carcinoma HCT 116 cell line using a 3D cell culture model. All these compounds individually inhibited viability, colony growth, and the invasion of 3D HCT 116 spheroids in a variable degree with greater activity of linckoside L1. Polysaccharide preparation in combination with linckoside L1 exerted synergism of anticancer effects through the inactivation of protein kinase B, followed by induction of apoptosis via the regulation of proapoptotic/antiapoptotic proteins balance. The obtained data open up prospects for the development of new therapeutic approaches for colorectal cancer treatment.

A gene encoded glycosylated enzyme, β-galactosidase from *Alteromonas* sp. QD01, was cloned and expressed in *Escherichia coli* by Li et al. [14] to study the potential of this enzyme in synthesis of prebiotic galacto-oligosaccharides, which can improve the intestinal flora and could have important applications in medicine. The galactosidase showed wide pH stability in the pH range of 6.0–9.5, which is suitable for lactose hydrolysis in milk. Most metal ions promoted its activity, especially Mn^2+^ and Mg^2+^. This enzyme exhibited high transglycosylation activity and can probably catalyze the formation of galacto-oligosaccharides from milk and lactose. These characteristics indicated that Gal2A may be ideal for producing these prebiotics and lactose-reducing dairy products.
